# Are Depot Antipsychotics Ever Self-Tapering? Exploring the Discontinuation of Long-Acting Injectable Psychosis Drugs and Those With Delayed Onset Through In Silico Modelling

**DOI:** 10.1093/schizbullopen/sgag037

**Published:** 2026-09-12

**Authors:** James R O’Neill, Samantha L McLean, David M Taylor, Mark A Horowitz

**Affiliations:** Faculty of Medicine and Health, University of Leeds, Leeds, LS2 9JT, United Kingdom; Leeds Partnership NHS Foundation Trust, Clifton House, Leeds, YO30 5RA, United Kingdom; School of Pharmacy, Optometry and Medical Sciences, University of Bradford, Bradford, BD7 1DP, United Kingdom; School of Pharmacy, Optometry and Medical Sciences, University of Bradford, Bradford, BD7 1DP, United Kingdom; Wolfson Centre for Applied Health Research, Bradford, BD9 6TB, United Kingdom; Faculty of Life Sciences and Medicine, Kings College London, London, WC2R 2LS, United Kingdom; South London and Maudsley NHS Foundation Trust, London, SE5 8AZ, United Kingdom; Faculty of Life Sciences and Medicine, Kings College London, London, WC2R 2LS, United Kingdom; North East London NHS Foundation Trust, London, IG3 8XJ, United Kingdom

**Keywords:** deprescribing, antipsychotic, tapering, receptor occupancy, modelling, psychosis

## Abstract

**Background and Hypothesis:**

Reducing and stopping antipsychotic medications can have longer-term positive outcomes for some patients. Despite common wisdom, abrupt discontinuation of several long-acting injectable (LAI) antipsychotic drugs results in rapid D_2_ occupancy reductions. We explored if abrupt discontinuation of LAI antipsychotics with slow clearance remained within specified constraints on rate of D_2_ occupancy change (RODOC), which might reduce risk of emerging symptoms of psychosis related to withdrawal or relapse.

**Study Design:**

Pharmacokinetic data were collated for 6 LAI antipsychotic forms (aripiprazole lauroxil, olanzapine pamoate, paliperidone palmitate 1-, 3-, and 6-monthly, and risperidone microspheres). Fifty-two studies comprising 6146 patients were included. In silico modelling was completed to predict RODOC from abrupt discontinuation. We used RODOC thresholds of 5 percentage-point (pp) difference per month as a proxy measure to theoretically estimate the likelihood of withdrawal effects emerging, with sensitivity analyses for 2.5 and 10 pp/month.

**Study Results:**

Abrupt cessation of LAIs is likely to exceed 5 pp/month RODOC for all examined LAIs except paliperidone palmitate 6-monthly. Peak rates of D_2_ occupancy change per month for an average person ranged from 5 pp/month (paliperidone palmitate 6-monthly) to 66.1 pp/month (risperidone microspheres). Significant interpersonal variation in pharmacokinetic factors was observed.

**Conclusions:**

Almost all LAI antipsychotic medications could theoretically cause withdrawal effects in most patients if abruptly discontinued without oral supplementation. However, abrupt discontinuation of longer-acting paliperidone palmitate could theoretically be successful for some patients. Prescribers must collaborate with patients when devising tapering regimens to ensure schedules are personalized and responsive to individual experience.

## Introduction

Reducing and discontinuing antipsychotics can have long-term positive effects on social functioning for some patients without worsening symptom severity.^1^^,^^2^ However, trials have shown increased rates of relapse with antipsychotic tapering.^1^^,^^3^ Consensual agreement is still lacking,^4^ but increasing focus is being placed on gradual and hyperbolic dose reduction to safely taper antipsychotic medications.^5^^,^^6^ The rate of a taper may play a role in determining relapse, rather than just reduction below a certain threshold of D_2_ dopamine receptor occupancy.^4^ Withdrawal effects from tapering too rapidly may precipitate a psychotic relapse.^7^

Slower tapering that corresponds to the hyperbolic pattern between dose and effect on target receptors may minimize withdrawal effects and reduce risk of relapse.^8–10^ Discontinuation of oral antipsychotics led to a 3-4-fold greater risk of relapse when compared to long-acting injectable (LAI) formulations at around 11 months follow-up.^11^ This could likely be attributed to LAI formulations being more slowly eliminated from the body.

However, it is also argued that relapse is provoked by dose reduction below a certain threshold,^12^ cited to be around 65% D_2_ receptor occupancy.^13^ It remains unclear whether receptor occupancy threshold or the rate at which tapering occurs (specifically regarding receptor occupancy) is the key factor.^14^

We have previously outlined guidance for clinicians regarding approaches to tapering LAI psychosis drugs in order to give consistent reductions in dopamine receptor occupancy. These included recommendations for aripiprazole monohydrate,^15^ as well as flupentixol, haloperidol, and zuclopenthixol decanoate.^16^ We examined the maximal rate of D_2_ receptor occupancy change (RODOC) from abrupt discontinuation to derive reduction regimens that observe certain rates of RODOC that may minimize risk of developing symptoms of psychosis.

Although LAI psychosis drugs have slower elimination from the body, control over plasma concentration and receptor occupancy when tapering is often more challenging than with oral forms. This has been observed in recent randomized controlled trials,^6^ where the only participants to experience worsening of symptoms during a 2-year proportionate reduction period were those prescribed LAI forms, even when gradually reduced in dose or prolonged in interval.

We have previously outlined the need for patients to invariably switch to oral medication at specified points during reduction regimens to limit rates of reduction in plasma concentration and D_2_ occupancy.^15^^,^^16^ This is especially important as drug concentration approaches the EC_50_ value (the concentration at which 50% of the maximum potential effect of a drug is observed). This is the “tipping point” at which RODOC is most rapid, resulting from the combined effects of drug elimination (exponential decay) and dose–receptor occupancy relationships (logarithmic decline at low doses).^15^ The amount of drug removed from the body per unit time through elimination reduces as overall plasma concentration. Conversely, changes in receptor occupancy are slow with decreases in drug at higher concentrations but progressively increase as plasma concentration reduces.^15–18^ These 2 relationships converge when considering rate of D_2_ occupancy change over time, and this convergence leads to the maximal rate of occupancy change manifesting around the point of EC_50_.

More recently, LAIs have been developed with slower apparent clearance due to delayed release characteristics, including paliperidone palmitate^19^ and aripiprazole lauroxil.^20^ Longer elimination half-life leads to slower rate of receptor occupancy change between administrations. Theoretically, it should be possible to abruptly discontinue a drug formulation if the rate of receptor occupancy changes while reducing through the EC_50_ value does not provoke emerging symptoms of psychosis related to withdrawal or relapse. This paper aims to establish whether current LAI drugs with slower or delayed clearance can be abruptly discontinued by examining rates of D_2_ receptor occupancy reduction.

## Methods

### Literature Search

A systematized literature search was proposed to identify relevant pharmacokinetic studies of 6 LAIs; monthly paliperidone palmitate (PP1M), quarterly paliperidone palmitate (PP3M), twice-yearly paliperidone palmitate (PP6M), olanzapine pamoate (OLAI), Risperdal Consta (RLAI), and aripiprazole lauroxil (AL).

Six databases were searched including Ovid MEDLINE, APA PsycInfo, Embase, International Pharmaceutical Abstracts, Clarivate Web of Science, and Scopus. Full search strategies can be seen in Supplementary Material 1.

Inclusion criteria were studies and case reports that completed therapeutic drug monitoring (TDM) of the above 6 drugs at steady-state (minimum of 4 injections prior to venous blood sample being obtained) in adult humans (aged over 18 years). Studies were excluded if timing of TDM samples was random or unspecified, as well as if concurrent oral forms of the same antipsychotic medication were prescribed. Only studies written in English were included for screening.

Other forms of risperidone LAI apart from Risperdal Consta, such as risperidone ISM,^21^ Rykindo,^22^ RBP-7000, and TV-46000 were not included in formal analysis. Moreover, the NanoCrystal Dispersion form of aripiprazole lauroxil^23^ was excluded from analysis due to not being a long-term maintenance form of administration.

Demographic (number of subjects, age, and sex) and pharmacokinetic data from included studies were extracted and collated on Microsoft Excel (version 2209, 64-bit, Redmond, WA, USA) software. Pharmacokinetic data of interest were maximal (*C*_max_) and minimal (*C*_min_) plasma drug concentrations during the inter-dose interval, as well as time to maximal concentration (*t*_max_), and apparent elimination half-life as a combination of drug release and clearance (*t*_1/2_). Mean and standard deviation values for the above were extracted if individual subject data were not available, allowing for inter-individual variation to be calculated across studies. For risperidone and paliperidone, active moiety concentrations were exclusively used.

### Pharmacokinetic Data Analysis

In silico modelling of plasma concentrations following a final LAI administration was undertaken using extracted pharmacokinetic data using Microsoft Excel software. Remaining proportions of drug were calculated at various intervals using a free-to-use online half-life calculator.^24^ Outputs obtained from this analysis included an “average” plasma concentration using median plasma concentrations and values for *t*_1/2_. Inter-percentile range thresholds of 10% and 90% for each of the pharmacokinetic parameters were used to generate models for “slow clearance” and “rapid clearance.” This approach has been utilized due to significant pharmacokinetic variation observed for several antipsychotics, including 5-fold for olanzapine^25^ and 20-fold for aripiprazole.^26^

Preliminary models were based on hypothetical patients who had been established (and therefore at “steady-state” concentrations) on the maximum licensed dosages for each individual LAI studied. While patients undergoing a tapering regimen would likely be on a lower maintenance dosage, maximum dosages were chosen to reflect the full range of how plasma concentration would translate to D_2_ occupancy over time. Maximal RODOC for each drug would remain consistent irrespective of the dose or dosing interval used for modelling. Time from discontinuation to breaching specified thresholds, a key aspect for clinical interpretation, is outlined in Supplementary Materials for both maximum licensing dosages, as well as the smallest currently available medicinal form of each drug.

### Dopamine Receptor Occupancy Analysis

Dopamine receptor occupancy was calculated using the Michaelis–Menten formula:


$$ \mathrm{Occupancy}={V_{\mathrm{max}}}^{\ast }\ \mathrm{concentration}/\left(\mathrm{concentration}+{\mathrm{EC}}_{50}\right) $$

where *V*_max_ is the determined maximal occupancy of receptors and EC_50_ is the plasma drug concentration required to generate 50% occupancy.

*V*_max_ and EC_50_ values for each drug were obtained from existing published PET imaging literature. Relationships were ascertained between D_2_ receptor occupancy and plasma concentrations for olanzapine^27^ and aripiprazole^28^ using 1 single PET study. Regarding receptor occupancy for the active moiety of risperidone and paliperidone, data from 3 PET studies^29–31^ were individually extracted and collated using non-linear regression modelling^32^ to devise *V*_max_ and EC_50_ parameters. Values for each of the drugs can be seen in Supplementary Table 1.

### RODOC Thresholds Used

The optimal RODOC to avoid symptoms of psychosis emerging is not known, and therefore, any chosen threshold for this remains theoretical on a population level until further empirical exploration. Notwithstanding this, our analysis of a trial of paliperidone LAI discontinuation seems to suggest that a RODOC of approximately 5 pp/month correlated with 50% of the cohort experiencing emergence of symptoms of psychosis.^33^ This was equivalent and consistent across both PP1M and PP3M groups, despite the latter being able to reduce to a much lower overall D_2_ occupancy threshold.

We have therefore used an arbitrary and theoretical threshold of 5 percentage points of RODOC to test reduction schedules against, with sensitivity analyses conducted for 2.5 and 10 pp of RODOC. These values have been used in previous similar studies^15^^,^^16^ due to them corresponding to periods often examined in discontinuation trials—which involve reductions over months to years—as well as the time taken for markers of neuro-adaptations to antipsychotics, like tardive dyskinesia, to resolve.^5^ Patients often report that tapering off antipsychotics can take long periods, with 23% of 1 cohort reporting it took them at least a year to taper off successfully.^34^

## Results

### Literature Search

There were 3321 publications initially obtained prior to de-duplication and screening. Forty-five articles met inclusion criteria, as did a further 7 obtained through reverse citation screening. Full results of the screening process can be seen in Supplementary Material 2.

Of the 52 included articles, 3 provided pharmacokinetic data for AL,^35–37^ 7 for OLAI,^27^^,^^38–43^ 12 for PP1M,^39^^,^^44–54^ 9 for PP3M,^39^^,^^51^^,^^52^^,^^55–60^ 3 for PP6M,^58^^,^^60^^,^^61^ and 24 for RLAI.^22^^,^^29–31^^,^^46^^,^^48^^,^^62–79^ Some publications included pharmacokinetic data for multiple drugs. Publications included in analysis for each drug are listed in Supplementary Material 3.

Six thousand one hundred forty-six subjects were included in analysis overall, with 122 for AL, 203 for OLAI, 2449 for PP1M, 1068 for PP3M, 477 for PP6M, and 1827 for RLAI. Full details of demographics for the sample are available in Supplementary Table 2.

### Pharmacokinetic Analysis

Pharmacokinetic parameters obtained and used to power modelling are listed in Table 1.

**Table 1:** Pharmacokinetic parameters obtained from literature search for the six drugs in question and used to power in silico modelling

Drug	Dose and dosing interval	Clearance status	Maximal steady-state concentration (*C* _max_, ng/ml)	Minimal steady-state concentration (*C* _min_, ng/ml)	Apparent elimination half-life (*t* _1/2_, days)
Aripiprazole lauroxil	882 mg every 4 weeks	Slow	509.3	490.9	94.3
		Average	293.1	268.1	38.9
		Rapid	110.0	85.3	13.6
Olanzapine pamoate	405 mg every 4 weeks	Slow	50.4	33.8	41.5
		Average	44.9	22.8	24.6
		Rapid	23.0	8.9	17.6
Paliperidone palmitate 1-monthly	150 mg every month	Slow	72.8	62.7	168.0
		Average	40.7	33.2	78.2
		Rapid	24.9	10.6	18.7
Paliperidone palmitate 3-monthly	525 mg every 3 months	Slow	65.9	47.7	128.5
		Average	56.6	37.3	83.4
		Rapid	58.5	30.0	62.3
Paliperidone palmitate 6-monthly	1000 mg every 6 months	Slow	119.7	60.2	153.5
		Average	76.0	25.3	95.7
		Rapid	25.9	5.5	67.7
Risperidone microspheres (Risperdal Consta)	50 mg every 2 weeks	Slow	95.4	45.0	19.1
		Average	74.2	32.0	7.2
		Rapid	61.7	14.7	3.2

Figure 1 demonstrates the anticipated change in D_2_ receptor occupancy over time from a final injection of each drug given to a patient at steady-state concentration with regular dosing intervals. The 6 graphs respectively depict AL 882 mg 4-weekly (Fig. 1a), OLAI 405 mg 4-weekly (Fig. 1b), RLAI 50 mg fortnightly (Fig. 1c), PP1M 150 mg monthly (Fig. 1d), PP3M 525 mg quarterly (Fig. 1e) and PP6M 1000 mg twice-yearly (Fig. 1f). A graph depicting respective plasma drug concentrations during the same period can be seen in Supplementary Materials 4-9.

**Figure 1:**
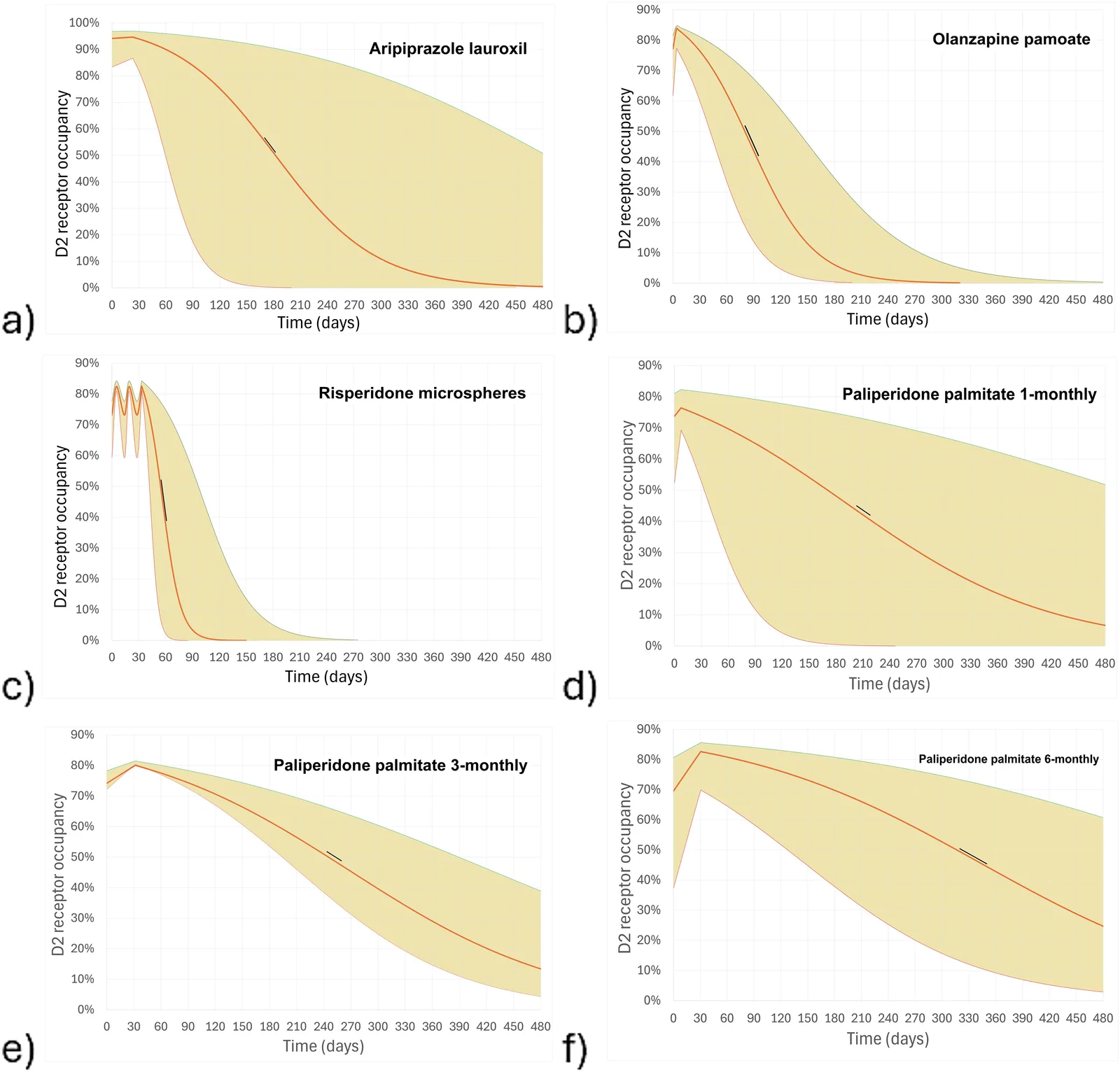
Graph Depicting the Change in D_2_ Receptor Occupancy Following Abrupt Discontinuation (Final Administration at *t* = 0 from Steady-State Plasma Concentrations) for a Patient Taking Various Long-Acting Injectable Antipsychotics. The green line depicts “slow” clearance, the orange line depicts a person with “average” clearance, and the red line depicts “rapid” clearance. The black line depicts the timepoint of maximal rate of D_2_ occupancy change for a person with “average” clearance. Drugs, dosages, and inter-dose intervals included are (a) 882 mg AL 4-weekly, (b) 405 mg OLAI 4-weekly, (c) 50 mg RLAI fortnightly, (d) 150 mg PP1M monthly, (e) 525 mg PP3M quarterly, and (f) 1000 mg PP6M twice-early.

### Aripiprazole Lauroxil

When considering a patient with “average” clearance, the peak rate of D_2_ occupancy change was 13.4 percentage points per month and occurred at approximately 6 months following the final LAI injection. The peak rate for “slow” clearance was 5.5 percentage points per month, whereas for “rapid” clearance, this was 38.3 points per month.

For a person with “average” clearance, it would take around 9 weeks before RODOC breached 5 percentage points per month. For the person with “slow” clearance, it would take around 7-8 months before they breached RODOC of 2.5 percentage points per month and around 13 months before they breached RODOC of 5 percentage points per month.

### Olanzapine Pamoate

When considering a person with “average” clearance, the peak rate of D_2_ occupancy change was 19.5 percentage points per month and occurred at approximately 3 months following the final LAI injection. The peak rate for “slow” clearance was 11.5 percentage points per month, whereas for “rapid” clearance, this was 27.2 points per month.

For a person with “average” clearance, RODOC would breach 10 percentage points by the end of the 4-week dosing interval. For the person with “slow” clearance, it would take around 5 weeks before they breached RODOC of 5 percentage points per month and around 4 months before they breached RODOC of 10 percentage points per month.

### Risperidone Microspheres (Risperdal Consta)

The key difference to account for with discontinuing RLAI is the delayed onset of the drug following administration. This leads to ongoing fluctuations in plasma concentration, and therefore D_2_ occupancy, for several weeks after the final administration.

When considering a person with “average” clearance, the peak rate of D_2_ occupancy change for RLAI was 66.1 percentage points per month and occurred at approximately 8 weeks following the final LAI injection. The peak rate for “slow” clearance was 24.8 percentage points per month, whereas for “rapid” clearance, this was 147.2 points per month.

For a person with “slow” clearance, it would take approximately 6 weeks from abrupt discontinuation until they experienced a greater RODOC than 10 percentage points per month.

### Paliperidone Palmitate 1-Monthly

When considering a person with “average” clearance, the peak rate of D_2_ occupancy change was 6.1 percentage points per month and occurred at approximately 6 months following the final LAI injection. The peak rate for “slow” clearance was 2.3 percentage points per month, whereas for “rapid” clearance, this was 25.4 points per month.

For a person with “average” clearance, it would take approximately 3 months from abrupt discontinuation until they experienced a faster RODOC than 5 percentage points per month. It would take around a year for a person with “slow” clearance to breach 2.5 percentage points RODOC per month.

### Paliperidone Palmitate 3-Monthly

When considering a person with “average” clearance, the peak rate of D_2_ occupancy change was 5.7 percentage points per month and occurred at approximately 8-9 months following the final LAI injection. The peak rate for “slow” clearance was 3.7 percentage points per month, whereas for “rapid” clearance, this was 7.6 points per month.

For a person with “average” clearance, it would take around 6 months before the RODOC was faster than 5 percentage points per month. It would take a similar duration of time before a person with “slow” clearance experienced RODOC greater than 2.5 percentage points per month.

### Paliperidone Palmitate 6-Monthly

When considering a person with “average” clearance, the peak rate of D_2_ occupancy change was exactly 5 percentage points per month and occurred at approximately 11 months following the final LAI injection. The peak rate for those with “slow” clearance was 3.1 percentage points per month, whereas for “rapid” clearance, this was 7 points per month. It would take a person with “slow” clearance around 14 months before they experienced RODOC greater than 2.5 percentage points per month.

### Time to Breaching Tolerable Threshold

Supplementary Table 3 demonstrates the duration following final administration of the abovementioned drugs before the rate of change in D_2_ occupancy would breach our pre-defined tolerability thresholds of 2.5, 5, and 10 percentage points per month. This has been articulated for both maximum licensing dosages, as well as the currently available minimum medicinal forms, given that these LAIs are invariably packaged in either single-use pre-filled syringes or provided with a specified powder and solvent.

The values in Supplementary Table 3 theoretically suggest an estimated timepoint at which a patient undergoing a tapering regimen may need to commence a low-dose oral form of the drug in order to possibly avoid or alleviate withdrawal effects. It may be preferable to switch such patients to oral medication prophylactically (if clinically reasonable) to allow a more gradual reduction over time.

### Plasma Concentration at Point of Breaching Threshold

Table 2 depicts the plasma concentration for each drug at the point when the rate of change in D_2_ occupancy exceeded our stipulated thresholds of 2.5, 5, and 10 percentage points per 30 days. This may, in principle, be used by clinicians as a theoretical guide alongside TDM to personalize plans for individual patients with regard to dose reduction and discontinuation.

**Table 2:** Minimal Plasma Concentration (*C*_min_) for Individual LAI Drugs at Which Rate of D_2_ Occupancy Change Would be Anticipated to Exceed 2.5, 5, or 10 Percentage Points per Month

Drug	Plasma concentration (ng/ml)		
	2.5 pp/month	5 pp/month	10 pp/month
AL	117.1	89.8	51.0
OLAI	n/a	30.5	16.3
PP1M	28.8	19.5	n/a
PP3M	42.4	16.6	n/a
PP6M	33.5	17.0	n/a

### LAI Dose Reduction as Means of Tapering

We have outlined theoretical principles for tapering 6 LAI formulations using the maximum licensing dosages for each. Of course, many patients will be prescribed lower dosages than this and may be able to tolerate lower maintenance doses without switching to oral forms. It should always be considered whether or not a patient’s dose can be reduced successfully while remaining in LAI form, but this needs to be determined and assessed on an individual basis. A potential tool for ascertaining this would be using TDM at the time of *C*_min_ for their current dose and then comparing results to parameters that we have outlined in Table 3 to suggest whether dose reduction while remaining in LAI form is likely to be theoretically viable.

**Table 3:** Suggested Guidelines for Commencing Switch to Oral Form of Medication Following Abrupt Discontinuation of Maximum Dose of LAI Form

Drug	Slow clearance	Average clearance	Rapid clearance
AL	Commence 2.5 mg aripiprazole 6 months after last LAI dose, continue for at least 12 months, before further 20% proportionate tapering on a monthly basis	Commence 3 mg aripiprazole 2 months after last LAI dose, continue for at least 9 months, before further 20% proportionate tapering on a monthly basis	Commence 5 mg aripiprazole when LAI would have next been due, continue for at least 3 months, before further 20% proportionate oral tapering on a monthly basis
OLAI	Commence 2 mg olanzapine 5 weeks after last LAI dose, continue for at least 6 months, before further 20% proportionate tapering on a monthly basis	Commence 2.5 mg olanzapine when LAI would have next been due, continue for at least 4 months, before further 20% proportionate tapering on a monthly basis	Commence 2.5 mg olanzapine when LAI would have next been due, continue for at least 3 months, before further 20% proportionate tapering on a monthly basis
PP1M	Commence 500 μg risperidone 6 months after last LAI dose, continue for at least 9 months, before further 20% proportionate tapering on a monthly basis	Commence 750 μg risperidone 3 months after last LAI dose, continue for at least 6 months, before further 20% proportionate tapering on a monthly basis	Commence 1 mg risperidone when LAI would have been next due, continue for at least 4 months, before further 20% proportionate tapering on a monthly basis
PP3M	Commence 250 μg risperidone 6 months after last LAI dose, continue for at least 12 months, before further 20% proportionate tapering on a monthly basis	Commence 500 μg risperidone 4 months after last LAI dose, continue for at least 12 months, before further 20% proportionate tapering on a monthly basis	Commence 750 μg risperidone when LAI would have been next due, continue for at least 12 months, before further 20% proportionate tapering on a monthly basis
PP6M	Should be able to tolerate abrupt discontinuation	Commence 250 μg risperidone 9 months after last LAI dose, continue for at least 12 months, before further 20% proportionate tapering on a monthly basis	Commence 250 μg risperidone when LAI would have been next due, continue for at least 15 months, before further 20% proportionate tapering on a monthly basis
RLAI	Commence 2 mg risperidone 6 weeks after last LAI dose, continue for 3 months, before further 20% proportionate tapering on a monthly basis	Commence 3 mg risperidone 6 weeks after last LAI dose, continue for 10 weeks, before further 20% proportionate tapering on a monthly basis	Commence 4 mg risperidone 6 weeks after last LAI dose, and conduct further 20% proportionate tapering on a monthly basis

If someone is prescribed a starting dose at *t* = 0 that is lower than the licensed maximum, the peak RODOC for the drug will still be the same as at a higher dose (assuming that elimination half-life is equivalent). However, the time at which this peak RODOC is experienced would be sooner after discontinuation. Supplementary Table 3 demonstrates the time difference to peak RODOC in days between the minimum and maximum licensed doses for each of the 6 forms explored.

To calculate the likely lowest viable dose while remaining on LAI form (assuming that breaching our threshold of 5 percentage points per month would correlate with withdrawal effects), the following calculation could be applied:


\begin{align*} & \mathrm{Target\ dose}\\& = \mathrm{(current\ dose/current\ concentration)}^{\ast }\ \mathrm{target\ concentration} \end{align*}

The concentration values outlined in Table 3 should be considered absolute minimums, and therefore, the calculated target dose should always be rounded up (rather than down) to the nearest available medicinal form.

To use a hypothetical example, we calculated that a person with “average” clearance taking PP1M 150 mg monthly would have a *C*_min_ of 33.2 ng/ml. Our findings suggest that they would theoretically be able to reduce their dose to that corresponding with a *C*_min_ of 19.5 ng/ml before they would experience excessive D_2_ variation during the inter-dose interval (again, assuming that 5 percentage points per month is the tolerable RODOC threshold). They would therefore, in theory, be able to reduce their dose to 88 mg ([150/33.2]*19.5) monthly, if such a dose could be practically administered. Therefore, an appropriate and practical alternative would be the next highest available medicinal form of 100 mg monthly. Once again, this recommendation depends inherently on the assumption that adhering to a dose reduction trajectory of no more than 5 percentage points of D_2_ occupancy per month corresponds with tapering without emergence of withdrawal-related symptoms or relapse; if a slower trajectory were required, a more cautious approach would of course be required.

Prolonging the dosing interval, however, could also be possible.^15^ Caution must be exercised by prescribers though, as this is more complex than simply assuming a linear relationship between dosing interval duration and concentration. In other words, doubling the inter-dose interval would not necessarily lead to the *C*_min_ being halved, as is the case with dose–concentration relationships. For the above example, we calculated that prolonging the inter-dose interval of PP1M 150 mg for a person with “average” clearance to 2-monthly would result in an anticipated *C*_min_ of 25.5 ng/ml. However, prolonging further to 3-monthly would result in an expected *C*_min_ of 19.5 ng/ml, which would, by our threshold (and the assumption that adhering to this would be clinically viable), just about still be tolerable.

We have outlined potential strategies for each of the 6 forms for each of the 3 clearance statuses in Supplementary Tables 4-6 with regard to reducing dose or prolonging the dosing interval, which should adhere to our recommended thresholds of not exceeding 2.5 (Supplementary Table 4), 5 (Supplementary Table 5), and 10 (Supplementary Table 6) percentage points of D_2_ occupancy change per month while maintaining LAI formulations.

### Supplementation With Oral Forms

We have outlined that, in line with our previous similar work^15^^,^^16^ and despite having much longer elimination half-lives that their oral counterparts, abrupt cessation is in theory unlikely to be tolerated well by patients for the vast majority of LAI formulations when attempting discontinuation. As a result, switching to an oral form of the same drug will likely be required to avoid or reduce unpleasant withdrawal effects.

Exactly when such a switch will be required will depend greatly upon individual pharmacokinetics, such as their clearance status. We have demonstrated through our modelling that vast interpersonal differences in pharmacokinetics occur across the population, which may explain the vast differences seen in tolerability of various tapering strategies. Consideration and mitigation for this should be undertaken by prescribers when advising tapering regimens. Therapeutic drug monitoring can be a useful tool for ascertaining a person’s clearance status,^80^ as can pharmacogenetic testing.^81^

The oral dose of medication required for such a switch will depend on a person’s clearance status (someone with quicker clearance would likely require a higher oral dose at an earlier stage in the schedule). However, it is important to account for continued presence of the LAI drug as this is being eliminated, and often only a very low oral dose (in some cases, accounting for as little as 15% D_2_ occupancy^27^) is, according to our calculations, likely to be required. What is equally essential is that this low dose is maintained for a prolonged period, likely for at least several months, while the LAI continues to be eliminated. Given that periods without medication might lead to disengagement from services, earlier commencement of oral antipsychotics might be prudent than strictly pharmacologically required.

A rough estimation for required oral switch doses for each drug is listed in Table 3, but we again stress that this should be amended and personalized in line with patient tolerability and TDM. Recommendations for the 3 clearance statuses are provided to indicate the potential ranges of suitable oral switch dosages and timepoints at which they should be commenced. Table 3 provides guidance for patients who are taking the maximum dose of the given depot—for those taking lower doses, RODOC thresholds will be reached earlier (see Supplementary Table 3), and commencing oral supplementation must therefore be undertaken at an earlier point.

Our recommendations are based upon the assumption that 1 mg oral risperidone equating to approximately 6 ng/ml active moiety,^82^ 1 mg oral olanzapine equating to a plasma concentration of 2.1 ng/ml,^83^ and 1 mg aripiprazole equating to a plasma concentration of 12.2 ng/ml.^15^ Oral tapering should be undertaken in line with previously published recommendations.^5^ Although the rate of D_2_ occupancy decline implies that delays may be possible in commencement of oral medication, it may be more practical clinically to commence oral supplementation soon after depot is stopped to prevent disengagement with treatment.

It is imperative to stress that the above recommendations are based on several assumptions, and might not be applicable for all patients trying to reduce or discontinue antipsychotics. Individualization of tapering regimens, ideally through TDM, is fundamental to optimizing the clinical success of any attempts at dose reduction, and the above guidance should not be simply followed in a “set-and-forget” manner without close monitoring and supervision.

## Discussion

### Adherence to Tolerable Thresholds

We found that the maximal rate of change in D_2_ occupancy for AL, RLAI, and OLAI exceeded our pre-determined threshold of 5 percentage points per month for all 3 clearance statuses. This suggests that abrupt discontinuation of these formulations may, in theory, be likely to precipitate symptoms of psychosis related to withdrawal or relapse. Patients would most likely need to switch to oral forms to allow for a graduated reduction regimen according to the findings of our modelling study.

For all 3 forms of paliperidone palmitate, those with “slow” clearance experienced a maximal rate of D_2_ occupancy change that was less than the same pre-determined threshold, and therefore some patients may be able to tolerate abrupt discontinuation without a need to switch to an oral form. However, for those with “average” or “rapid” clearance, the maximal rate of change exceeded our threshold for all paliperidone forms except PP6M.

### Wide Pharmacokinetic Variation

Our review of existing pharmacokinetic literature for several LAIs suggests there to be significant interpersonal variation. This has been previously reported for several other psychotropic drugs; flupentixol half-life has been found to vary by 6-7 fold,^84^ while sertraline plasma concentrations can vary by 18-19 fold across the population.^85^

Although pharmacokinetic variation has been accounted for in this study, there will, of course, be outlier patients who clear the drug faster or slower than the limits of our analysis. Some patients will experience withdrawal effects from abrupt discontinuation of PP6M. Similarly, some patients may be able to tolerate abrupt discontinuation of shorter-acting LAIs, just as a minority of patients may not experience adverse effects from abrupt cessation or oral medications.

This is why individualized tapering regimens that account for a person’s previous or anticipated response to dose reduction are fundamental. Also, any proposed tapering plan must be adaptable based on the patient’s experience. Therapeutic drug monitoring or pharmacogenetic study can be highly beneficial in ascertaining a person’s clearance status to personalize tapering regimens.

### Acceptable Threshold for Rate of D_2_ Occupancy Change

There continues to be debate around whether a “therapeutic threshold” for antipsychotics exists (below which efficacy would abruptly decrease) or whether it is the rate at which receptor occupancy changes that provokes emergence of psychotic symptoms. Supplementary Table 7 outlines the timepoint at which abrupt discontinuation of each LAI drug for each clearance status would, according to our calculations, lead to D_2_ receptor occupancy reducing below the cited 65% occupancy threshold for therapeutic efficacy. The discrepancy between these timepoints and our findings for when RODOC is greatest may elucidate further on whether the “threshold” or “velocity” models are more pertinent.

If rate of tapering is indeed a key factor in risk of withdrawal effects, then it is important to keep the rate of receptor occupancy change linear throughout a taper, and for this to take place slowly and gradually. However, it as yet remains unclear what such rate of change in D_2_ occupancy should be targeted during a tapering regimen in order to avoid provoking antipsychotic withdrawal symptoms. This no doubt varies greatly between individuals, at least when medication dosages and frequencies are considered in isolation, but whether such variability can be explained solely through interpersonal pharmacokinetic differences in drug clearance remains to be ascertained.

Through this study, we have chosen previously utilized RODOC thresholds of 2.5, 5, and 10 percentage point differences per month in this study. As stated earlier, these remain theoretical until further empirical validation. However, a study comparing time-to-relapse between oral and LAI medication found that the 3-monthly form of paliperidone palmitate (PP3M) had a delayed time-to-relapse when compared to 1-monthly (PP1M); both LAI forms had longer time-to-relapse than abrupt discontinuation of oral forms.^33^ Moreover, the timepoint at which 50% of participants in this study relapsed correlated with a lower anticipated plasma concentration (and therefore D_2_ receptor occupancy) for PP3M (with a longer elimination half-life) than PP1M. This lends support to the “velocity” hypothesis, in that rate of change (calculated to be around 5 percentage points D_2_ occupancy per month in the abovementioned trial^33^ for both LAI forms) may be more predictive of emergence of psychosis than reduction below a specified threshold of D_2_ occupancy (calculated to be around 50% on average for PP1M compared to approximately 25% for PP3M).

A recent small-scale study of inter-dose interval symptom variation found that patients could tolerate variation of around 15 percentage points per month without experiencing any observable change in mental state.^86^ However, this study was very short-term and did not assess reduction in dose, only the inter-dose interval between steady-state *C*_max_ and *C*_min_. In addition, the findings of this study conflict with the abovementioned trial,^33^ where abrupt PP3M discontinuation (which our modelling suggests to cause peak RODOC for an average person of 5.5 percentage points per month) led to over 40% of the sample relapsing within a year. Similar findings were observed in the HAMLETT trial, where hyperbolic dose reductions over a 6-month period (corresponding to between 10 and 15 percentage points per month, if reductions are kept perfectly consistent throughout) still resulted in increased relapse rates and reduced quality of life.^1^ However, it is noted that the final stages of the reductions employed by this trial still resulted in significant abrupt changes in D_2_ receptor occupancy, which may have precipitated the negative outcomes in the short term.^87^

The more conservative thresholds that this study has employed also align with anecdotal clinical experience that tapering regimens typically need to be undertaken over at least a 2-year period to have the greatest chance of being successful. However, it is worth acknowledging that duration of treatment with antipsychotic medications will likely place a significant role in ascertaining how quickly or slowly tapering can take place. Longer-term users of antipsychotic medications will likely need to taper at a slower rate due to a greater degree of postsynaptic D_2_ receptor upregulation, and duration of antipsychotic use should be reflected in the considered speed of tapering.

As well as duration of treatment, clinicians should note that reduction and discontinuation of partial D_2_ agonists such as aripiprazole may be better tolerated than full antagonist medications, as has been found in a recent trial.^88^ This may be due to reduced effects of dopamine receptor upregulation, which might minimize the risk of super-sensitivity psychosis. This may mean that tapering of these medications can take place at a quicker rate, but a quantifiable value for this remains to be determined. Further real-world studies of our theoretical recommendations, as well as comparisons of differing tapering schedules, will be informative.

### Limitations

In this study, in silico modelling utilized previous pharmacokinetic studies (invariably focussed around the introduction and maintenance of a drug). These were extrapolated in order to estimate the effects of discontinuation with regard to changes in plasma concentrations and D_2_ occupancy. While some previous pharmacokinetic studies have explored dosing interval prolongation for LAI drugs such as risperidone,^31^^,^^67^ further pharmacokinetic exploration of LAI prolongation, reduction, and discontinuation is required in order to strengthen the confidence that can be placed in our findings.

Furthermore, it is well established that psychosis drugs have a far broader pharmacodynamic effect than on D_2_ receptors alone. This includes effects on the serotonergic^89^ and histaminergic^90^^,^^91^ monoamine systems. Therefore, when considering a person’s tolerability to discontinuing psychotropics, the rate of change at all target receptors during a proposed regimen should be considered in order to ensure that withdrawal effects do not manifest. Unfortunately, there is no available research that has analysed the dose–occupancy relationship between LAI antipsychotics and other receptors aside from D_2_ receptors. It may also be difficult, given the lack of clarity around a tolerable rate of change at D_2_ receptors, to distinguish between whether withdrawal effects have manifested due to dopaminergic withdrawal or due to a different monoamine system. This requires further research and exploration in the future.

Pharmacokinetic studies vary in utilizing mean or median values for describing pharmacokinetic parameters depending on the distribution of their datasets. Our datasets for the 6 drugs were invariably found to be not normal (see Supplementary Table 8 for further details), hence our use of median values and inter-percentile ranges. While a sensitivity analysis of our datasets using mean values for pharmacokinetic parameters (see Supplementary Table 9) found largely comparable findings in terms of whether or not discontinuation of a drug would result in RODOC being breached, there were subtle differences in the resulting recommendations and strategies for tapering.

This highlights yet again not only the vast interpersonal variation in population pharmacokinetics of these drugs and the need to individualize tapering regimens but also how further empirical data could help to improve the accuracy of our recommendations and the need for clinicians to take a cautious approach incorporating close observation when implementing these in practice.

## Conclusions

Abrupt cessation of LAI medications may result in excessive rate of change in dopamine receptor occupancy for a person with “average” clearance. This in theory may risk psychotic symptoms emerging and a tapering regimen failing and so would not be supported on the basis of our findings. Some patients will be able to reduce their dose and prolong their inter-dose interval with cautious pharmacokinetic monitoring. A minority of people should, theoretically, be able to abruptly discontinue longer acting forms of paliperidone palmitate.

Further research is still needed around tapering psychotropics, not least LAI forms. This may strengthen the evidence base for hyperbolic, or proportionate, tapering, as well as further elucidation around the balance between timely dose reduction and ensuring patient tolerability.

The guidance that we have outlined provides a theoretical starting point for clinicians, as well as points of consideration for prescribers and patients to be aware of when planning a dose reduction. Notwithstanding this, we observed vast interpersonal variation in pharmacokinetic factors, further emphasizing the need for personalized, bespoke, and responsive tapering, which takes into account a person’s previous successes or difficulties in reducing psychotropic medication. Therapeutic drug monitoring or pharmacogenomic testing may identify those who could tolerate quicker reductions, as well as those who may require a slower, more conservative approach.

## Supplementary Material

longer_LAI_discontinuation_supp_materials_sgag037
