# Supplementary material for: Are Depot Antipsychotics Ever Self-Tapering? Exploring the Discontinuation of Long-Acting Injectable Psychosis Drugs and Those With Delayed Onset Through In Silico Modelling
Source: Schizophr Bull Open. 2026 Sep 12;7(1):sgag037. doi: 10.1093/schizbullopen/sgag037 (PMC13644859; doi:10.1093/schizbullopen/sgag037)
Supplement: Supplementary file 1 [file longer_lai_discontinuation_supp_materials_sgag037.docx]

*Are depot antipsychotics ever self-tapering? Exploring the discontinuation of long-acting injectable psychosis drugs and those with delayed onset*

James R. O’Neill

Samantha L. McLean

David M. Taylor

Mark A. Horowitz

**SUPPLEMENTARY MATERIALS**

Supplementary Material 1: Search strategies for the six databases included	2
Supplementary Material 2: Flow diagram of systematic literature search	8
Supplementary Material 3: Articles used for pharmacokinetic analysis	9
Supplementary Material 4: Graph showing plasma concentration for AL	16
Supplementary Material 5: Graph showing plasma concentration for OLAI	17
Supplementary Material 6: Graph showing plasma concentration for PP1M	18
Supplementary Material 7: Graph showing plasma concentration for PP3M	19
Supplementary Material 8: Graph showing plasma concentration for PP6M	20
Supplementary Material 9: Graph showing plasma concentration for RLAI	21
Supplementary Table 1: Values for Michaelis-Menten receptor occupancy equations	22
Supplementary Table 2: Demographics of sample	23
Supplementary Table 3: Time difference to peak RODOC for max. and min. dosages	24
Supplementary Table 4: Recommended strategies adhering to 2.5 pp RODOC	25
Supplementary Table 5: Recommended strategies adhering to 5 pp RODOC	26
Supplementary Table 6: Recommended strategies adhering to 10 pp RODOC	27
Supplementary Table 7: Time until <65% D2 receptor occupancy	28
Supplementary Table 8: Tests for normal distribution of datasets	29
Supplementary Table 9: Sensitivity analysis using mean values from dataset	30

**Supplementary Material 1: Search strategies for the six databases included**

Ovid MEDLINE(R) ALL <1946 to April 03, 2025>

1	((olanzapine and (embonate or pamoate or depot or LAI or long-acting)) or “zyprexa adhera” or zypadhera).tw,kf.	432

2	Olanzapine/	6512

3	Delayed-Action Preparations/	42432

4	2 and 3	140

5	1 or 4	469

6	(((risperdal or rispolept or risperidon?) and (consta or microspher* or microparticl* or depot or "long acting" or long-acting or lai)) or okedi).ti,ab,tw.	973

7	risperidone/ and delayed-action preparations/	443

8	6 or 7	1053

9	((aripiprazole and lauroxil) or aristada).tw,kf.	82

10	aripiprazole/ and delayed-action preparations/	163

11	9 or 10	213

12	((paliperidone and (palmitate or long-acting or depot or LAI)) or (Invega AND (sustenna or trinza or hafyera)) or xeplion or byannli or trevicta).tw,kf.	834

13	paliperidone/ and delayed-action preparations/	359

14	12 or 13	962

15	5 or 8 or 11 or 14	2034

16	(pharmacokinetic* or bioavailab* or half-li#e or absor* or tmax or cmax or cmin or distribut* or metaboli* or excret*).tw,kf.	3817825

17	exp Pharmacokinetics/	346302

18	16 or 17	3961963

19	15 and 18	443

APA PsycInfo <1806 to March 2025 Week 5>

1	((olanzapine and (embonate or pamoate or depot or LAI or long-acting)) or "zyprexa adhera" or zypadhera).ti,ab,tw.	316

2	olanzapine/ and injections/	45

3	1 or 2	316

4	(((risperdal or rispolept or risperidon?) and (consta or microspher* or microparticl* or depot or "long acting" or long-acting or lai)) or okedi).ti,ab,tw.	714

5	risperidone/ and injections/	148

6	4 or 5	719

7	((aripiprazole and lauroxil) or aristada).ti,ab,tw.	39

8	aripiprazole/ and injections/	35

9	7 or 8	68

10	((paliperidone and (palmitate or long-acting or depot or LAI)) or (Invega and (sustenna or trinza or hafyera)) or xeplion or byannli or trevicta).ti,ab,tw.	469

11	(pharmacokinetic* or bioavailab* or half-li#e or absor* or tmax or cmax or cmin or distribut* or metaboli* or excret*).ti,ab,tw.	225101

12	exp pharmacokinetics/	3417

13	11 or 12	225798

14	3 or 6 or 9 or 10	1176

15	13 and 14	184

Embase Classic+Embase <1947 to 2025 April 03>

1	((olanzapine and (embonate or pamoate or depot or LAI or long-acting)) or "zyprexa adhera" or zypadhera).tw,kf	897

2	olanzapine/ and long acting drug/	287

3	1 or 2	1095

4	(((risperdal or rispolept or risperidon?) and (consta or microspher* or microparticl* or depot or "long acting" or long-acting or lai)) or okedi).tw,kf	2153

5	risperidone/ and long acting drug/	489

6	4 or 5	2348

7	((aripiprazole and lauroxil) or aristada).tw,kf	178

8	aripiprazole lauroxil/	232

9	7 or 8	243

10	((paliperidone and (palmitate or long-acting or depot or LAI)) or (Invega and (sustenna or trinza or hafyera)) or xeplion or byannli or trevicta).tw,kf	1869

11	paliperidone/ and long acting drug/ 263

12	10 and 11	1994

13	(pharmacokinetic* or bioavailab* or half-li#e or absor* or tmax or cmax or cmin or distribut* or metaboli* or excret*).tw,kf	4836401

14	exp pharmacokinetics/	912526

15	13 or 14	5306705

14	3 or 6 or 9 or 12	4040

15	13 and 14	1038

International Pharmaceutical Abstracts <1970 to March 2025>

1	((olanzapine and (embonate or pamoate or depot or LAI or long-acting)) or "zyprexa adhera" or zypadhera).ti,ab,tw.	89

2	olanzapine/	2114

3	injections/	14528

4	2 and 3	40

5	1 or 4	107

6	(((risperdal or rispolept or risperidon?) and (consta or microspher* or microparticl* or depot or "long acting" or long-acting or lai)) or okedi).ti,ab,tw.	288

7	risperidone/ and injections/	107

8	6 or 7	308

9	((aripiprazole and lauroxil) or aristada).ti,ab,tw.	22

10	aripiprazole lauroxil/	24

11	9 or 10	26

12	((paliperidone and (palmitate or long-acting or depot or LAI)) or (Invega and (sustenna or trinza or hafyera)) or xeplion or byannli or trevicta).ti,ab,tw.	201

13	paliperidone palmitate/	146

14	12 or 13	217

15	(pharmacokinetic* or bioavailab* or half-li#e or absor* or tmax or cmax or cmin or distribut* or metaboli* or excret*).ti,ab,tw.	138871

16	pharmacokinetics/	72374

17	15 or 16	151747

18	5 or 8 or 11 or 14	523

19	17 and 18	156

Web of Science Search Strategy (search conducted on April 03, 2025)

\# Database: Web of Science Core Collection

\# Entitlements:

Search: #6 AND #5	Date Run: Fri Apr 04 2025 16:07:37 GMT+0100 (British Summer Time)	Results: 539

Search: #1 OR #2 OR #3 OR #4	Date Run: Fri Apr 04 2025 16:07:15 GMT+0100 (British Summer Time)	Results: 3103

Search: TS=(pharmacokinetic* or bioavailab* or half-li?e or absor* or tmax or cmax or cmin or distribut* or metaboli* or excret*)	Date Run: Fri Apr 04 2025 16:04:45 GMT+0100 (British Summer Time)	Results: 8797848

Search: TS=((paliperidone and (palmitate or long-acting or depot or LAI)) or (Invega AND (sustenna or trinza or hafyera)) or xeplion or byannli or trevicta)	Date Run: Fri Apr 04 2025 16:04:08 GMT+0100 (British Summer Time)	Results: 1392

Search: TS=((aripiprazole and lauroxil) or aristada)	Date Run: Fri Apr 04 2025 16:03:40 GMT+0100 (British Summer Time)	Results: 103

Search: TS=(((risperdal or rispolept or risperidon$) and (consta or microspher* or microparticl* or depot or "long acting" or long-acting or lai)) or okedi)	Date Run: Fri Apr 04 2025 16:02:36 GMT+0100 (British Summer Time)	Results: 1845

Search: olanzapine and (embonate or pamoate or depot or LAI or long-acting) or “zyprexa adhera” or zypadhera (Topic)	Date Run: Fri Apr 04 2025 15:58:42 GMT+0100 (British Summer Time)	Results: 623

Scopus Search Strategy (search conducted on April 03, 2025)

1. TITLE-ABS-KEY ( ( olanzapine AND ( embonate OR pamoate OR depot OR lai OR long-acting ) ) OR "zyprexa adhera" OR zypadhera )	1236
2. TITLE-ABS-KEY ( ( ( risperdal OR rispolept OR risperido* ) AND ( consta OR microspher* OR microparticl* OR depot OR "long acting" OR long-acting OR lai ) ) OR okedi )	2196
3. TITLE-ABS-KEY ( ( aripiprazole AND lauroxil ) OR aristada )	201
4. TITLE-ABS-KEY ( ( paliperidone AND ( palmitate OR long-acting OR depot OR lai ) ) OR ( invega AND ( sustenna OR trinza OR hafyera ) ) OR xeplion OR byannli OR trevicta )	1656
5. #1 or #2 or #3 or #4	3468
6. TITLE-ABS-KEY ( pharmacokinetic* OR bioavailab* OR half-li?e OR absor* OR tmax OR cmax OR cmin OR distribut* OR metaboli* OR excret* )	14411145
7. #5 AND #6	961

**Supplementary Material 2: Flow diagram of systematic literature search**

**Identification of studies via databases and registers**

3321 records identified from*:

Databases (n = 6)

Embase (n = 1038)

OVID MedLine (n = 443)

APA PsycInfo (n = 184)
Scopus (n = 961)

Web of Science (n = 539)

Ovid IPA (n = 156)

Records removed *before screening*:

Duplicate records removed (n = 1630)

**Identification**

Records excluded (n = 1385)

No pharmacokinetic data (n = 765)

Wrong drug (n = 277)
Not LAI formulation (n = 216)
Not human-based (n = 112)
PK data not at steady-state (n = 26)

Records screened at title, abstract and subject heading level

(n = 1691)

**Screening**

Reports not retrieved

(n = 3)

Reports sought for retrieval

(n = 306)

Reports excluded (n = 258):

No original data / duplicated study (n = 85)

No pharmacokinetic data (n = 66)
Wrong drug (n = 36)

Insufficient data on dose/drug (n = 33)

TDM timing not appropriate / unclear (n = 10)

Not LAI formulation (n = 9)

Not in English / no translation available (n = 6)

Not human-based (n = 5)

PK data not at steady-state (n = 5)

Not in vivo (n = 3)

Reports assessed for eligibility

(n = 303)

Reports included (n = 52)

**Included**

Reports obtained through reverse citation screening (n = 7)

**Supplementary Material 3: Articles incorporated in pharmacokinetic data analysis for individual drugs**

*Aripiprazole lauroxil*

| First author | Year | Title | Journal |
| --- | --- | --- | --- |
|  |  |  |  |
| Alkermes Inc. | 2025 | Aristada (aripirazole lauroxil) | n/a |
| Center for Drug Evaluation and Research | 2015 | Clinical pharmacology and biopharmaceutics review of aripiprazole lauroxil (NDA 207533) | n/a |
| Weiden | 2020 | Pharmacokinetics, Safety, and Tolerability of a 2‑Month Dose Interval Regimen of the Long‑Acting Injectable Antipsychotic Aripiprazole Lauroxil: Results From a 44‑Week Phase I Study | CNS Drugs |

*Olanzapine pamoate*

| First author | Year | Title | Journal |
| --- | --- | --- | --- |
| Baldelli | 2018 | Intraindividual and Interindividual Variability of Olanzapine Trough Concentrations in Patients Treated With the Long-Acting Injectable Formulation | Journal of Clinical Psychopharmacology |
| D'Anna | 2022 | Therapeutic Drug Monitoring of Long-Acting Injectable Antipsychotics as a Predictor of Relapse in Schizophrenia Spectrum Disorders: A 1-Year Pilot Study | Therapeutic Drug Monitoring |
| Kurtz | 2008 | Pharmacokinetics (PK) of Multiple Doses of Olanzapine Long Acting Injection (OLAI), an Intramuscular (IM) Depot Formulation of Olanzapine (OLZ), in Stabilized Patients with Schizophrenia | Biological Psychiatry |
| Mamo | 2008 | D2 Receptor Occupancy of Olanzapine Pamoate Depot Using Positron Emission Tomography: An Open-label Study in Patients with Schizophrenia | Neuropsychopharmacology |
| Manouilenko | 2018 | Long-acting olanzapine injection during pregnancy and breastfeeding: a case report | Archives of Women's Mental Health |
| Mauri | 2015 | "Long-acting" olanzapine in maintenance therapy of schizophrenia: A study with plasma levels | International Journal of Psychiatry Clinical Practice |
| Mitchell | 2013 | Single- and Multiple-Dose Pharmacokinetic, Safety, and Tolerability Profiles of Olanzapine Long-Acting Injection: An Open-Label, Multicenter, Nonrandomized Study in Patients With Schizophrenia | Clinical Therapeutics |
|  |  |  |  |
|  |  |  |  |

*Paliperidone palmitate 1-monthly*

| First author | Year | Title | Journal |
| --- | --- | --- | --- |
| Chiu | 2023 | Therapeutic Drug Monitoring of Paliperidone in Patients With Schizophrenia in Taiwan | Journal of Clinical Pharmacology |
| Coppola | 2012 | A one-year prospective study of the safety, tolerability and pharmacokinetics of the highest available dose of paliperidone palmitate in patients with schizophrenia | BMC Psychiatry |
| D'Anna | 2022 | Therapeutic Drug Monitoring of Long-Acting Injectable Antipsychotics as a Predictor of Relapse in Schizophrenia Spectrum Disorders: A 1-Year Pilot Study | Therapeutic Drug Monitoring |
| Gopal | 2011 | A Post-hoc Comparison of Paliperidone Palmitate to Oral Risperidone During Initiation of Long-acting Risperidone Injection in Patients with Acute Schizophrenia | Innovations in Clinical Neuroscience |
| Hough | 2009 | Safety and tolerability of deltoid and gluteal injections of paliperidone palmitate in schizophrenia | Progress in Neuro-Psychopharmacology & Biological Psychiatry |
| Hyza | 2021 | Plasma Levels of Long-Acting Injectable Antipsychotics in Outpatient Care: A Retrospective Analysis | Neuropsychiatric Disease and Treatment |
| Rossenu | 2015 | Pharmacokinetic Profile After Multiple Deltoid or Gluteal Intramuscular Injections of Paliperidone Palmitate in Patients With Schizophrenia | Clinical Pharmacology in Drug Development |
| Russu | 2019 | Pharmacokinetic-Pharmacodynamic Characterization of Relapse Risk for Paliperidone Palmitate 1-Month and 3-Month Formulations | Journal of Clinical Psychopharmacology |
| Samtani | 2016 | Prospective dose selection and acceleration of paliperidone palmitate 3‐month formulation development using a pharmacometric bridging strategy | British Journal of Clinical Pharmacology |
| Savitz | 2016 | Efficacy and Safety of Paliperidone Palmitate 3-Month Formulation for Patients with Schizophrenia: A Randomized, Multicenter, Double-Blind, Noninferiority Study | International Journal of Neuropsychopharmacology |
| Schoretsanitis | 2022 | Effects of body weight, smoking status, and sex on plasma concentrations of once-monthly paliperidone palmitate | Expert Review of Clinical Pharmacology |
| Shimizu | 2019 | Population Pharmacokinetics of Paliperidone Palmitate (Once-Monthly Formulation) in Japanese, Korean, and Taiwanese Patients With Schizophrenia | Clinical Pharmacology in Drug Development |

*Paliperidone palmitate 3-monthly*

| First author | Year | Title | Journal |
| --- | --- | --- | --- |
| Berwaertz | 2015 | Efficacy and Safety of the 3-Month Formulation of Paliperidone Palmitate vs Placebo for Relapse Prevention of Schizophrenia: A Randomized Clinical Trial | JAMA Psychiatry |
| Bois | 2024 | A Bayesian framework for virtual comparative trials and bioequivalence assessments | Frontiers in Pharmacology |
| D'Anna | 2022 | Therapeutic Drug Monitoring of Long-Acting Injectable Antipsychotics as a Predictor of Relapse in Schizophrenia Spectrum Disorders: A 1-Year Pilot Study | Therapeutic Drug Monitoring |
| Fernández-Miranda | 2023 | Plasmatic Levels and Response to Variable Doses of Monthly Aripiprazole and Three-Month Paliperidone in Patients with Severe Schizophrenia. Treatment Adherence, Effectiveness, Tolerability, and Safety | Neuropsychiatric Disease and Treatment |
| Najarian | 2021 | A Randomized, Double-Blind, Multicenter, Noninferiority Study Comparing Paliperidone Palmitate 6-Month Versus the 3-Month Long-Acting Injectable in Patients With Schizophrenia | International Journal of Neuropsychopharmacology |
| Samtani | 2016 | Paliperidone Palmitate 3-Month Dosing in Special Patient Populations and Derivation of Secondary Pharmacokinetic Parameters Based on Simulations | Biological Psychiatry |
| Samtani | 2016 | Prospective dose selection and acceleration of paliperidone palmitate 3‐month formulation development using a pharmacometric bridging strategy | British Journal of Clinical Pharmacology |
| Savitz | 2016 | Efficacy and Safety of Paliperidone Palmitate 3-Month Formulation for Patients with Schizophrenia: A Randomized, Multicenter, Double-Blind, Noninferiority Study | International Journal of Neuropsychopharmacology |
| T'jollyn | 2024 | Model-Informed Clinical Development of 6-Monthly Injection of Paliperidone Palmitate in Patients with Schizophrenia: Dosing Strategies Guided by Population Pharmacokinetic Modeling and Simulation (Part II) | European Journal of Drug Metabolism |

*Paliperidone palmitate 6-monthly*

| First author | Year | Title | Journal |
| --- | --- | --- | --- |
| T'jollyn | 2024 | Model-Informed Clinical Development of 6-Monthly Injection of Paliperidone Palmitate in Patients with Schizophrenia: Dosing Strategies Guided by Population Pharmacokinetic Modeling and Simulation (Part II) | European Journal of Drug Metabolism |
| Najarian | 2021 | A Randomized, Double-Blind, Multicenter, Noninferiority Study Comparing Paliperidone Palmitate 6-Month Versus the 3-Month Long-Acting Injectable in Patients With Schizophrenia | International Journal of Neuropsychopharmacology |
| Janssen Pharmaceuticals | 2025 | Invega Hafyera® (paliperidone palmitate) extended-release injectable suspension, for gluteal intramuscular use | n/a |
|  |  |  |  |
|  |  |  |  |

*Risperdal® Consta®*

| First author | Year | Title | Journal |
| --- | --- | --- | --- |
| Bai | 2007 | Pharmacokinetics study for hyperprolactinemia among schizophrenics switched from risperidone to risperidone long-acting injection | Journal of Clinical Psychopharmacology |
| Castberg | 2005 | Serum Concentrations of Risperidone and 9-Hydroxyrisperidone After Administration of the Long-Acting Injectable Form of Risperidone: Evidence From a Routine Therapeutic Drug Monitoring Service | Therapeutic Drug Monitoring |
| Choong | 2013 | Pharmacogenetic Study on Risperidone Long-Acting Injection: Influence of Cytochrome P450 2D6 and Pregnane X Receptor on Risperidone Exposure and Drug-Induced Side-Effects | Journal of Clinical Psychopharmacology |
| Eerdekens | 2004 | Pharmacokinetics and tolerability of long-acting risperidone in schizophrenia | Schizophrenia Research |
| Ganoci | 2021 | ABCB1, ABCG2 and CYP2D6 polymorphism effects on disposition and response to long-acting risperidone | Progress in Neuro-Psychopharmacology & Biological Psychiatry |
| Gefvert | 2005 | Pharmacokinetics and D2 receptor occupancy of long-acting injectable risperidone (Risperdal Consta) in patients with schizophrenia | International Journal of Neuropsychopharmacology |
| Gharabawi | 2007 | Maintenance therapy with once-monthly administration of long-acting injectable risperidone in patients with schizophrenia or schizoaffective disorder: a pilot study of an extended dosing interval | Annals of General Psychiatry |
| Gopal | 2011 | A Post-hoc Comparison of Paliperidone Palmitate to Oral Risperidone During Initiation of Long-acting Risperidone Injection in Patients with Acute Schizophrenia | Innovations in Clinical Neuroscience |
| Green | 2015 | Long-acting injectable vs oral risperidone for schizophrenia and co-occurring alcohol use disorder: a randomized trial | Journal of Clinical Psychiatry |
| Hendset | 2009 | Impact of CYP2D6 Genotype on Steady-State Serum Concentrations of Risperidone and 9-Hydroxyrisperidone in Patients Using Long-Acting Injectable Risperidone | Journal of Clinical Psychopharmacology |
| Hyza | 2021 | Plasma Levels of Long-Acting Injectable Antipsychotics in Outpatient Care: A Retrospective Analysis | Neuropsychiatric Disease and Treatment |
| Ikai | 2012 | A Cross-Sectional Study of Plasma Risperidone Levels With Risperidone Long-Acting Injectable: Implications for Dopamine D2 Receptor Occupancy During Maintenance Treatment in Schizophrenia | Journal of Clinical Psychiatry |
| Lai | 2009 | Pharmacokinetics and efficacy of a direct switch from conventional depot to risperidone long-acting injection in Chinese patients with schizophrenic and schizoaffective disorders | Psychiatry & Clinical Neurosciences |
| Ma | 2021 | Effects of CYP2D6, CYP3A5, and ABCB1 gene polymorphisms on the pharmacokinetics of two risperidone long-acting injection microsphere formulations | Progress in Neuro-Psychopharmacology & Biological Psychiatry |
| Medori | 2006 | Plasma antipsychotic concentration and receptor occupancy, with special focus on risperidone long-acting injectable | European Neuropsychopharmacology |
| Meltzer | 2014 | A six month randomized controlled trial of long acting injectable risperidone 50 and 100 mg in treatment resistant schizophrenia | Schizophrenia Research |
| Nesvag | 2006 | Serum concentrations of risperidone and9-OH risperidone following intramuscular injection of long-acting risperidone compared with oral risperidone medication | Acta Psychiatrica Scandinavica |
| Peng | 2008 | The Disparity of Pharmacokinetics and Prolactin Study for Risperidone Long-Acting Injection | Journal of Clinical Psychopharmacology |
| Remington | 2006 | A PET Study Evaluating Dopamine D2 Receptor Occupancy for Long-Acting Injectable Risperidone | American Journal of Psychiatry |
| Thyssen | 2010 | Risperidone long-acting injection: pharmacokinetics following administration in deltoid versus | Journal of Clinical Psychopharmacology |
| Uchida | 2008 | Monthly Administration of Long-Acting Injectable Risperidone and Striatal Dopamine D2 Receptor Occupancy for the Management of Schizophrenia | Journal of Clinical Psychiatry |
| Volonteri | 2010 | Long-acting injectable risperidone and metabolic ratio: a possible index of clinical outcome in treatment-resistant schizophrenic patients | Psychopharmacology |
| Walling | 2025 | Pharmacokinetics and safety of a novel extended-release microsphere formulation of Risperidone in Patients with Schizophrenia or Schizoaffective Disorder | Journal of Clinical Pharmacology |
| Wang | 2024 | Population Pharmacokinetic Analysis to Support and Facilitate Switching from Risperidone Formulations to Rykindo in Patients with Schizophrenia | Neurology and Therapy |
|  |  |  |  |

**Supplementary Material 4: Graph depicting the changes in plasma aripiprazole concentration for a patient taking 882mg aripiprazole lauroxil four-weekly following a final administration. Green line depicts a ‘slow’ clearance, orange line depicts an ‘average’ clearance, and red line depicts a ‘rapid’ clearance.**

**Supplementary Material 5: Graph depicting the changes in plasma olanzapine concentration for a patient taking 405mg olanzapine pamoate four-weekly following a final administration. Green line depicts a ‘slow’ clearance, orange line depicts an ‘average’ clearance, and red line depicts a ‘rapid’ clearance.**

**Supplementary Material 6: Graph depicting the changes in plasma active moiety (risperidone plus 9-hydroxyrisperidone) concentration for a patient taking 150mg paliperidone palmitate monthly following a final administration. Green line depicts a ‘slow’ clearance, orange line depicts an ‘average’ clearance, and red line depicts a ‘rapid’ clearance.**

**Supplementary Material 7: Graph depicting the changes in plasma active moiety (risperidone plus 9-hydroxyrisperidone) concentration for a patient taking 525mg paliperidone palmitate 3-monthly following a final administration. Green line depicts a ‘slow’ clearance, orange line depicts an ‘average’ clearance, and red line depicts a ‘rapid’ clearance.**

**Supplementary Material 8: Graph depicting the changes in plasma active moiety (risperidone plus 9-hydroxyrisperidone) concentration for a patient taking 1000mg paliperidone palmitate 6-monthly following a final administration. Green line depicts a ‘slow’ clearance, orange line depicts an ‘average’ clearance, and red line depicts a ‘rapid’ clearance.**

**Supplementary Material 9: Graph depicting the changes in plasma active moiety (risperidone plus 9-hydroxyrisperidone) concentration for a patient taking 50mg Risperdal Consta fortnightly following a final administration. Green line depicts a ‘slow’ clearance, orange line depicts an ‘average’ clearance, and red line depicts a ‘rapid’ clearance.**

**Supplementary Table 1: Values used to calculate dopamine receptor occupancy for each drug**

| **Drug** | **V_max_ (%)** | **EC_50_ (ng/ml)** |
| --- | --- | --- |
| Aripiprazole | 100.208 | 17.24 |
| Olanzapine | 92.02 | 4.39 |
| Active moiety (risperidone/paliperidone) | 91.21 | 7.89 |

**Supplementary Table 2: Demographics of the subjects included in pharmacokinetic analysis for each drug**

| Drug | n | Age, years, mean (SD) | Male, % |
| --- | --- | --- | --- |
| AL | 122 | 44.8 (10.2) | 73.8 |
| OLAI | 203 | 38.5 (10.2) | 65.0 |
| PP1M | 2449 | 39.8 (12.0) | 56.4 |
| PP3M | 1068 | 40.0 (12.7) | 57.7 |
| PP6M | 477 | 41.2 (11.8) | 68.1 |
| RLAI | 1827 | 40.3 (11.7) | 59.8 |

**Supplementary Table 3: Time from final administration (t=0) of LAI drug form until peak RODOC observed for a person with ‘average’, and how this differs between the minimum recommended and maximal licensing dosages for each form**

|  | | MINIMUM LICENSED DOSE | | MAXIMUM LICENSED DOSE | |
| --- | --- | --- | --- | --- | --- |
|  | Peak RODOC (pp/month) | Dose and inter-dose interval | **Time to peak RODOC (weeks)** | Dose and inter-dose interval | **Time to peak RODOC (weeks)** |
| AL | 13.4 | 441mg four-weekly | **19.5** | 882mg four-weekly | **25** |
| OLAI | 19.5 | 210mg fortnightly | **11** | 300mg fortnightly | **12.5** |
| PP1M | 6.1 | 25mg monthly | **4 (end of inter-dose interval)** | 150mg monthly | **26** |
| PP3M | 5.7 | 175mg three-monthly | **17** | 525mg three-monthly | **35.5** |
| PP6M | 5.0 | 700mg six-monthly | **41** | 1000mg six-monthly | **48** |
| RLAI | 66.1 | 25mg fortnightly | **7** | 50mg fortnightly | **8** |

**Supplementary Table 4: Recommended strategies for LAI dose reduction or prolongation of the inter-dose interval which should not breach our threshold of 2.5 percentage points per month. These strategies account for currently available medicinal forms of each drug.
RLAI is not listed as abrupt discontinuation would likely not be permissible for any clearance status. Similarly, those with ‘rapid’ clearance would be unlikely to tolerate abrupt discontinuation of any LAI, and are therefore also not listed.**

|  | ‘Slow’ clearance | ‘Average’ clearance |
| --- | --- | --- |
| AL | No scope for LAI dose reduction or prolongation of dosing interval | No scope for LAI dose reduction or prolongation of dosing interval |
| OLAI | No scope for LAI dose reduction or prolongation of dosing interval | No scope for LAI dose reduction or prolongation of dosing interval |
| PP1M | Prolong the inter-dose interval to no longer than three months (not applicable for 25mg dosages) | No scope for LAI dose reduction or prolongation of dosing interval |
| PP3M | Prolong the inter-dose interval to no longer than six months | No scope for LAI dose reduction or prolongation of dosing interval |
| PP6M | Prolong the inter-dose interval to no longer than twelve months | No scope for LAI dose reduction or prolongation of dosing interval |

**Supplementary Table 5: Recommended strategies for LAI dose reduction or prolongation of the inter-dose interval which should not breach our threshold of five percentage points per month. These strategies account for currently available medicinal forms of each drug.
RLAI is not listed as abrupt discontinuation would likely not be permissible for any clearance status.**

|  | ‘Slow’ clearance | ‘Average’ clearance | ‘Rapid’ clearance |
| --- | --- | --- | --- |
| AL | Reduce to 441mg and prolong inter-dose interval no longer than six MONTHS | Reduce to 662mg and prolong inter-dose interval no longer than six WEEKS | No scope for LAI dose reduction or prolongation of dosing interval |
| OLAI | Prolong the inter-dose interval no longer than five WEEKS | No scope for LAI dose reduction or prolongation of dosing interval | No scope for LAI dose reduction or prolongation of dosing interval |
| PP1M | Could tolerate abrupt discontinuation | Reduce to 100mg and prolong inter-dose interval no longer than six WEEKS | No scope for LAI dose reduction or prolongation of dosing interval |
| PP3M | Could tolerate abrupt discontinuation | Reduce to 350mg and prolong inter-dose interval no longer than four MONTHS | No scope for LAI dose reduction or prolongation of dosing interval |
| PP6M | Could tolerate abrupt discontinuation | Reduce to 700mg and prolong inter-dose interval no longer than nine months | No scope for LAI dose reduction or prolongation of dosing interval |

**Supplementary Table 6: Recommended strategies for LAI dose reduction or prolongation of the inter-dose interval which should not breach our threshold of ten percentage points per month. These strategies account for currently available medicinal forms of each drug.
RLAI is not listed as abrupt discontinuation would likely not be permissible for any clearance status. Conversely, PP3M and PP6M are not listed as both should be permissible with abrupt discontinuation for all three clearance statuses.**

|  | ‘Slow’ clearance | ‘Average’ clearance | ‘Rapid’ clearance |
| --- | --- | --- | --- |
| AL | Could tolerate abrupt discontinuation | Reduce to 441mg and prolong inter-dose interval to every two MONTHS | No scope for LAI dose reduction or prolongation of dosing interval |
| OLAI | Reduce to 300mg and prolong the inter-dose interval no longer than two MONTHS | No scope for LAI dose reduction or prolongation of dosing interval | No scope for LAI dose reduction or prolongation of dosing interval |
| PP1M | Could tolerate abrupt discontinuation | Could tolerate abrupt discontinuation | No scope for LAI dose reduction or prolongation of dosing interval |

**Supplementary Table 7: Time in days from abrupt discontinuation of LAI until D2 receptor occupancy reduces below 65% threshold**

| Drug | ‘Slow’ clearance | ‘Average’ clearance | ‘Rapid’ clearance |
| --- | --- | --- | --- |
| AL | 400 | 148 | 48 |
| OLAI | 98 | 56 | (occupancy would reduce below 65% during inter-dose interval) |
| PP1M | 326 | 90 | (occupancy would reduce below 65% during inter-dose interval) |
| PP3M | 257 | 159 | 130 |
| PP6M | 431 | 218 | (occupancy would reduce below 65% during inter-dose interval) |
| RLAI | 77 | 48 | (occupancy would reduce below 65% during inter-dose interval) |

**Supplementary Table 8: Results of Jarque-Bera tests of dataset normal distribution**

| Drug | Skewness | Excess kurtosis | Χ^2^ | p |
| --- | --- | --- | --- | --- |
| AL | -0.24 | 1.19 | 10.36 | 0.01 |
| OLAI | 0.32 | 1.01 | 26.18 | <0.01 |
| PP1M | 0.28 | 0.21 | 36.49 | <0.01 |
| PP3M | 2.10 | 6.97 | 1454.45 | <0.01 |
| PP6M | 0.02 | -0.20 | 204.69 | <0.01 |
| RLAI | 0.74 | 14.91 | 10356.32 | <0.01 |

**Supplementary Table 9: Sensitivity analysis using mean values from dataset of time from final administration (t=0) of LAI drug form until peak RODOC observed for a person with ‘average’ clearance, and how this differs between the minimum recommended and maximal licensing dosages for each form.**

|  | | MINIMUM LICENSED DOSE | | MAXIMUM LICENSED DOSE | |
| --- | --- | --- | --- | --- | --- |
|  | Peak RODOC (pp/month) | Dose and inter-dose interval | **Time to peak RODOC (weeks)** | Dose and inter-dose interval | **Time to peak RODOC (weeks)** |
| AL | 11.1 | 441mg four-weekly | **26** | 882mg four-weekly | **31.5** |
| OLAI | 16.7 | 210mg fortnightly | **11** | 300mg fortnightly | **13.5** |
| PP1M | 6.4 | 25mg monthly | **4.5 (end of inter-dose interval)** | 150mg monthly | **30** |
| PP3M | 5.5 | 175mg three-monthly | **24** | 525mg three-monthly | **43** |
| PP6M | 5.0 | 700mg six-monthly | **43** | 1000mg six-monthly | **48** |
| RLAI | 38.4 | 25mg fortnightly | **8** | 50mg fortnightly | **10** |
